# Olfactory training enhances semantic verbal fluency in healthy older adults, but only for individuals with low baseline performance

**DOI:** 10.1093/chemse/bjaf032

**Published:** 2025-09-05

**Authors:** Marta Rokosz, Sabina Barszcz, Michał Pieniak, Łukasz Gargula, Daniel Marek, Paulina Nawrocka, Aleksandra Reichert, Barbara Żyżelewicz, Maciej Barański, Katarzyna Resler, Anna Oleszkiewicz

**Affiliations:** Institute of Psychology, University of Wroclaw, Wroclaw, Poland; Institute of Psychology, University of Wroclaw, Wroclaw, Poland; Institute of Psychology, University of Wroclaw, Wroclaw, Poland; Interdisciplinary Center Smell & Taste, Department of Otorhinolaryngology, Faculty of Medicine Carl Gustav Carus, Technische Universität Dresden, Dresden, Germany; Institute of Psychology, University of Wroclaw, Wroclaw, Poland; Institute of Psychology, University of Wroclaw, Wroclaw, Poland; Institute of Psychology, University of Wroclaw, Wroclaw, Poland; Institute of Psychology, University of Wroclaw, Wroclaw, Poland; Interdisciplinary Center Smell & Taste, Department of Otorhinolaryngology, Faculty of Medicine Carl Gustav Carus, Technische Universität Dresden, Dresden, Germany; Institute of Psychology, University of Wroclaw, Wroclaw, Poland; Institute of Psychology, University of Wroclaw, Wroclaw, Poland; Department and Clinic of Otolaryngology, Head and Neck Surgery, Wroclaw Medical University, ul. Borowska 213, Wroclaw 50-556, Poland; Institute of Psychology, University of Wroclaw, Wroclaw, Poland; Interdisciplinary Center Smell & Taste, Department of Otorhinolaryngology, Faculty of Medicine Carl Gustav Carus, Technische Universität Dresden, Dresden, Germany

**Keywords:** olfactory training, olfaction, semantic verbal fluency, working memory, aging

## Abstract

Olfactory training (OT), a structured exposure to odors, is commonly used by otorhinolaryngologists to treat olfactory dysfunction. However, OT has been shown to improve cognition of people with cognitive or olfactory impairments and slow the age-related cognitive decline. This study investigated whether OT could enhance cognitive functions in older adults with an intact sense of smell, compared with younger adults. We hypothesized that OT would improve semantic verbal fluency and working memory in the experimental group, with no significant changes in the placebo group (PG). The final sample comprised 184 participants aged 24 to 94 years (*M*_age_ = 51.84 ± 23.25 years), including 83 young adults (53% women, *M*_age_ = 26.71 ± 2.62 years) and 101 older adults (88% women, *M*_age_ = 72.49 ± 5.40 years). For the semantic verbal fluency task, they listed as many items as possible within one of 2 semantic categories: (i) grocery products or (ii) fragrant items, within a 60-s time limit. To measure working memory, participants completed a digit span task where they repeated sequences of digits aloud. In older adults performing OT, it led to a marginal increase in semantic verbal fluency, regardless of semantic category, while no significant changes were observed in the older PG or in young adults. This effect was mainly driven by individuals with lower scores at baseline. Our findings suggest that OT can benefit semantic verbal fluency in the healthy geriatric population with lower baseline scores, but these results urge similar testing in clinical groups with compromised verbal functions.

## 1. Introduction

Olfactory system exhibits remarkable plasticity and susceptibility to training (Hummel et al. 2018). Olfactory training (OT) is a treatment method based on the structured, systematic exposure to odors, that otorhinolaryngologists use to rehabilitate olfactory dysfunction (Pieniak et al. 2022). Studies have demonstrated that OT can improve olfactory abilities, typically measured by the odor detection threshold, odor discrimination, and odor identification tests in patients with olfactory dysfunctions of various etiologies (for a systematic review and meta-analyses, see Delgado-Lima et al. 2024). OT has been shown to slow down the decline of olfactory functions with aging (Schriever et al. 2014).

Emerging evidence demonstrates that OT effectiveness may exceed olfaction and extend to cognitive abilities and emotional well-being (Wegener et al. 2018; Oleszkiewicz et al. 2021a; Vance et al. 2024; Pieniak et al. 2025). Preliminary evidence suggests that OT can slow age-related cognitive decline in older adults (Schriever et al. 2014; Oleszkiewicz et al. 2021a). OT has also been found to enhance semantic verbal fluency in adults and older adults, both with dysosmia and normosmia (Wegener et al. 2018; Oleszkiewicz et al. 2022). A short-term, intensive OT program enhanced cognitive domains of attention, memory, and language, reflected in an increase in verbal fluency as well as recognition, recalling, and memorization of words in patients with dementia (Cha et al. 2022). Finally, patients with mild cognitive impairments showed a slight increase in global cognition, following a standard OT regimen (Chen et al. 2022). It is worth noting that the presented studies have focused on participants with decreased either olfactory or cognitive functioning and the observed effects served as a form of rehabilitation.

Interestingly, even smaller, but still intentional odor exposure can influence cognitive functioning, both positively and negatively (for a review, see Johnson 2011). Exposure to different odorants modulated low-level visual perception, with participants perceiving slower motions of moving dots with a lemon odorant, and faster with vanilla (Tsushima et al. 2021). Presenting high-valence scents such as peppermint and cinnamon to drivers increased their alertness and decreased temporal demand (Raudenbush et al. 2009). Unpleasant smells can impair cognition, with the smell of ethyl mercoptan impairing complex tasks such as proofreading, although not simple tasks, such as arithmetic (Rotton 1983).

There are several reasons for expecting positive effects of OT on cognition in humans and explanations to why we observe these effects (review in Vance et al. 2024). In the medical field, there is a large body of research showing the connection between poor olfaction and decline in cognitive functioning. Olfactory loss often precedes neurodegenerative diseases, such as Parkinson's (Haehner et al. 2019) and Alzheimer's disease (Jung et al. 2019). Poor olfactory performance has been associated with increased future risk of dementia in otherwise cognitively healthy older adults (Yaffe et al. 2017). These observations reflect in anatomical brain structure and physiological connections between olfaction and cognition. Signals from the olfactory bulb to higher cortical areas partially bypass the thalamus, directly transferring olfactory stimuli to the amygdala-hippocampal complex (Freiherr 2017; Leon and Woo 2018). Neuroimaging studies confirm that OT influences the structure of the brain, for instance, by increasing the initially decreased volume of the orbitofrontal cortex, thalamus, and hippocampus in hyposmic patients (Gellrich et al. 2018). OT has also been shown to enhance functional connectivity in olfaction and cognition-related brain regions, by increasing a number of functional connections between these structures (Kollndorfer et al. 2015; Hosseini et al. 2020).

This study aimed to test whether OT can improve cognitive functions in older adults with an intact sense of smell and refer these findings to a group of young adults. We included commonly used in OT research measures of verbal fluency (Wegener et al. 2018; Cha et al. 2022; Oleszkiewicz et al. 2022) and working memory (Wegener et al. 2018). We expected OT to improve semantic verbal fluency and working memory in the experimental group, while no such change would be noted in the placebo group (PG). Additionally, we were interested whether the influence of OT on verbal fluency is domain-specific. One study has shown that olfactory memory training led to transfer into the untrained visual memory task (Olofsson et al. 2020). Thus, we expected that the potential OT effect on verbal fluency would be stronger in an odor-related category. We also assumed that the beneficial effect of OT in the older adult group was more likely to occur than in the young adult group, given that cognitive functions decline in older age, but are at their peak in early adulthood. Thus, the young adult group served as a reference category.

## 2. Materials and methods

### 2.1 Ethical statements

This study was conducted in line with the principles of the Declaration of Helsinki. The study design was approved by the Ethics Committee at the Institute of Psychology, University of Wroclaw (2021/YBKOC). The participants provided written consent to participate in the study and were informed that their consent could be withdrawn at any time.

### 2.2 Participants

Within a repeated-measures design with within × between factors interaction, to detect a moderate effect size of *f* = 0.25 (Oleszkiewicz et al. 2018) with a significance level of *α* = 0.05 and a statistical power of 0.95, a sample size of at least 68 participants per age group (136 in total) was estimated by G*Power software (Faul et al. 2007). Anticipating a dropout among the participants due to the longitudinal design of the study, we recruited 231 respondents (69% women) aged 24 to 94 years (*M*_age_ = 49.43 ± 23.63 years) from 2 age groups: young adults aged 24 to 38 years (52% women, *n*_1_ = 116; *M*_age_ = 26.67 ± 2.59 years) and older adults aged 64 to 94 years (88% women, *n*_2_ = 115; *M*_age_ = 73.0 ± 5.74 years). Participants from both age groups were randomly assigned to either an OT group (OTG) or a PG. There were 28 participants (17 adults) who did not return for the second measurement. For those who return for the post-training measurement, the exclusion criteria were: (i) <50% of OT compliance according to the monitoring app (more details in the Section 2.3) or paper-and-pencil diary for those participants who preferred this method over mobile app (adult group: *n* = 2; older group: *n* = 21) and (ii) neurological or olfactory health problems declared during the standardized medical interview (Welge-Lüssen et al. 2013). Based on these, we additionally excluded 19 respondents from the analyses. The final sample included 184 participants aged 24 to 94 (72% women, *M*_age_ = 51.84 ± 23.25 years), with 83 young adults (53% women, *M*_age_ = 26.71 ± 2.62 years) and 101 older adults (88% women, *M*_age_ = 72.49 ± 5.40 years). Detailed descriptives are shown in Table 1.

**Table 1.** Descriptives of OTG and PG across young adults and older adults groups.

	Adults			Older adults		
	OTG (*n* = 45)	PG (*n* = 38)	*t*/*χ*^2^	OTG (*n* = 50)	PG (*n* = 51)	*t*/*χ*^2^
***n* women**	21	23	*χ* ^2^(1) = 3.2, *P* = 0.21	44	45	*χ* ^2^(1) = 0.01, *P* = 0.94
**Age**	*M* = 27.0 ± 3.0	*M* = 26.4 ± 2.0	*t*(81) = 0.9, *P* = 0.36, *d* = 0.20	*M* = 72.0 ± 5.5	*M* = 72.9 ± 5.4	*t*(99) = 0.86, *P* = 0.39, *d* = 0.17
**OT compliance (%)**	*M* = 79.7 ± 12.90	*M* = 79.0 ± 13.7	*t*(81) = 0.3, *P* = 0.80, *d* = 0.06	*M* = 93.2 ± 10.1	*M* = 93.4 ± 11.0	*t*(99) = 0.11, *P* = 0.91, *d* = 0.02
	*M* = 79.4 ± 13.2			*M* = 93.3 ± 10.5		*t*(182) = 7.95, *P* < 0.001, *d* = 1.18

### 2.3 Procedure

Prior to being included in the study, the participants underwent a standardized medical interview to gather information about factors that might impair their sense of smell, such as diabetes, smoking, or current infections (Welge-Lüssen et al. 2013). The participants were tested twice, pre- and post-training. During the pretest, all participants completed tasks measuring working memory and semantic verbal fluency. Following this, participants were randomly assigned to one of the 2 groups. In the OTG, following the standard OT regimen, the participants smelled 4 odors: lemon (citronellal), eucalyptus (eucalyptol), rose (phenyl ethyl alcohol), and cloves (eugenol) (Hummel et al. 2009; Pieniak et al. 2022). Odors were delivered using Sniffin' Sticks, pen-like felt-tips odor dispensers (Hummel et al. 1997). The PG received sticks filled with odorless propylene glycol and was informed the odors were presented in subliminal concentration. Both groups were instructed to perform OT continuously for 12 weeks, twice a day (in the morning and the afternoon) (Hummel et al. 2009). A dedicated mobile application installed on the participants' cell phones reminded them about the OT and was used to monitor their OT compliance. The application ensured correct training execution by randomizing the order of odors within each OT session (to prevent fatigue and boredom resulting from the repetitiveness of the OT) and counting down the 20 s of exposure to each of the odors (or odorless sticks). The OT regimen included 2 control questions on the intensity and pleasantness of each odor using a graphical slider. These were included to maintain attention on the olfactory task and monitor potential odor spills or odor decay. If an odor was rated as unpleasant or not intense at all for at least 3 OT sessions, the experimental team would contact the participant to offer a new OT kit. The perceptual ratings of odors were not analyzed. After completion of OT, participants returned for a post-test, repeating the working memory tasks and semantic verbal fluency. Participants in the PG group were debriefed and informed they were smelling odorless pens for comparison purposes.

### 2.4 Measures

#### 2.4.1 Working memory

The Digit Span task assesses attention, concentration, and working memory for numbers. It is widely used in intelligence assessment, such as in the Wechsler Adult Intelligence Scale IV (WAIS-IV) and its earlier versions (Valentine et al. 2020). In our study, the researcher read aloud a sequence of random digits, starting with 3 digits and adding 1 digit with each subsequent reading. The longest prepared sequence of digits contained 11 elements. The participant then had to repeat the sequence in the same order. The task was untimed and continued until the participant made an error. The score was determined by the number of correctly repeated sequences, with a possible score ranging from 0 to 9 points (<3 to 11 elements in sequence, respectively). The second measurement was conducted similarly, but with a different order of the digits, to prevent participants from recalling the number sequences from the first measurement (i.e. learning effect).

#### 2.4.2 Semantic verbal fluency

Verbal fluency is a specific verbal ability, encompassing word retrieval from the mental lexicon, associated with access to this lexicon and with executive control processes (Shao et al. 2014). Verbal fluency was measured within a single task inspired by Benton and colleagues (Patterson 2011). We have asked the participants to list as many items as possible in one of 2 semantic categories: (i) grocery products or (ii) fragrant items. Time was restricted to 60 s. The participants were randomly assigned to one of the semantic categories, regardless of whether they were in the OTG or PG. Each participant answered the same question in both the pre- and post-test. The participant then verbally listed words, and the researcher used a finger counter to count the spoken words. The participant's score was the number of different words correctly listed in response to the given question within the allotted time. Higher score denotes more items listed and thus better performance in the task.

### 2.5 Statistical analyses

We analyzed data using R Statistical Software v. 4.4.1 (R Core Team 2024) with a significance level set to *P* < 0.05. First, we aimed to ensure that the drop-out was random and unrelated to the variables of interest. To this end, we compared participants who completed the OT with people who did not come back for the second measurement, using Welch's *t*-test.

To verify the effects of OT on working memory and semantic verbal fluency, we conducted a repeated-measures ANOVA (RMA). The within-subject factor was the time (pretraining vs post-training), and the between-subject factors were group (OTG vs PG) and age group (young adults vs older adults). For the semantic verbal fluency, we also added a between-subject factor of semantic category (fragrant vs grocery shop items). Group scores pre- and post-training are estimated marginal means ± standard error.

To verify if the observed significant effects of OT are a product of the regression to the mean effect, we conducted 3 additional analyses: (i) we ran an analysis of covariance (ANCOVA) model with the post-test value as dependent variable, experimental group (OTG, PG) and semantic category (grocery, fragrant items) as between-subject effects, and with the pretest score as a covariate (Senn 2006), (ii) the Blomqvist value (Blomqvist 1977), and (iii) the reliable change index (RCI)—whenever the index is >1.96, the change is classified as reliable (Jacobson and Truax 1991). As the latter formula requires a test–retest reliability coefficient, we used a test–retest correlation obtained in a study using semantic fluency task in healthy older participants (*M*_age_ = 67.7, standard deviation [SD] = 6.6, *r*_retest_ = 0.85) (Cooper et al. 2004).

## 3. Results

Adult participants who completed both measurements did not differ at the baseline measurement in terms of age, *t*(59.74) = 0.25, *P* = 0.801, *d* = 0.05, working memory, *t*(56.67) = 1.03, *P* = 0.305, *d* = 0.21, or semantic verbal fluency, *t*(69.41) = 0.03, *P* = 0.978, *d* = 0.01, from those who did not return for the second measurement or were excluded. In the older adults group, there were no differences in terms of working memory, *t*(12.64) = 1.03, *P* = 0.323, *d* = 0.30 and semantic verbal fluency, *t*(12.94) = 1.89, *P* = 0.081, *d* = 0.57 when compared with those who dropped out from the study or were excluded. However, those excluded from the analyses were older (*M* = 77.73 ± 6.81) than the final sample (*M*_age_ = 72.49 ± 5.40, *t*(11.41) = 2.47, *P* = 0.030, *d* = 0.85).

### 3.1 OT does not benefit working memory for digits

**Fig. 1.**
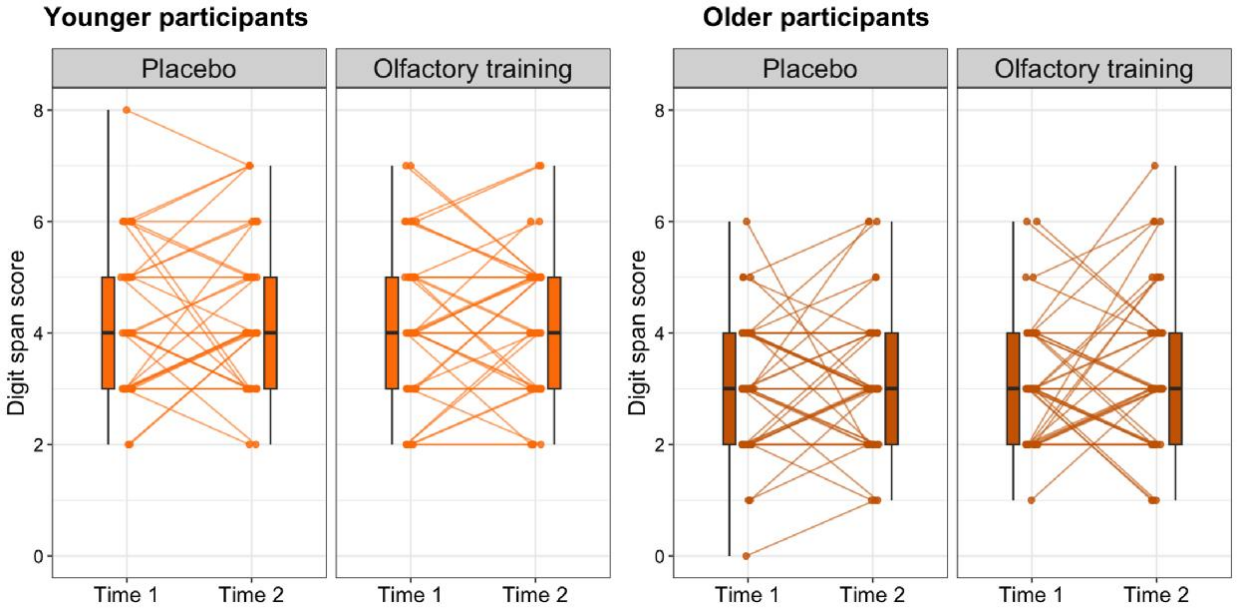
Digit span scores in OT and PGs at pre- and post-training. OTG, olfactory training group; PG, placebo group.

The RMA analysis revealed that there was no significant main effect of time of measurement (*F*_1,180_ = 0.80, *P* = 0.372, $\eta_{\mathrm{partial}}^{2}0.004$) or experimental group (*F*_1,180_ = 0.09, *P* = 0.770, $\eta_{\mathrm{partial}}^{2}<0.001$) on working memory. However, there was a significant main effect of age group (*F*_1,180_ = 46.17, *P* < 0.001, $\eta_{\mathrm{partial}}^{2}=0.204$). Adults (*M* = 4.20 ± 0.12) compared with older adults (*M* = 3.13 ± 0.11) memorized more sequences of digits. Interactions of time × group (*F*_1,180_ < 0.01, *P* = 0.977, $\eta_{\mathrm{partial}}^{2}<0.001$), time × age (*F*_1,180_ = 0.01, *P* = 0.909, $\eta_{\mathrm{partial}}^{2}<0.001$), group × age (*F*_1,180_ = 0.47, *P* = 0.494, $\eta_{\mathrm{partial}}^{2}=0.003$), and time × group × age group (*F*_1,180_ = 0.34, *P* = 0.56, $\eta_{\mathrm{partial}}^{2}=0.002$) were not significant. The results are presented in Fig. 1. Descriptive statistics for all variables of interest are presented in Table 2.

**Table 2.** Descriptive statistics of digit span and verbal fluency scores, presented by group.

	Adults						Older adults					
	OTG			PG			OTG			PG		
	T1 *M* (SE)	T2 *M* (SE)	*n*	T1 *M* (SE)	T2 *M* (SE)	*n*	T1 *M* (SE)	T2 *M* (SE)	*n*	T1 *M* (SE)	T2 *M* (SE)	*n*
**Digit span**	4.11 (0.18)	4.13 (0.19)	45	4.21 (0.20)	4.34 (0.21)	38	3.08 (0.17)	3.24 (0.18)	50	3.08 (0.17)	3.12 (0.18)	51
**VF—fragrant items**	18.14 (1.59)	20.71 (1.73)	21	16.60 (1.63)	17.85 (1.77)	20	14.09 (1.36)	17.35 (1.58)	23	15.56 (1.30)	15.60 (1.52)	25
**VF—grocery shop items**	33.75 (1.49)	35.13 (1.62)	24	28.11 (1.72)	30.11 (1.86)	18	23.89 (1.25)	25.41 (1.46)	27	23.46 (1.28)	24.31 (1.49)	26

M, estimated marginal mean; SE, standard error; VF, verbal fluency.

### 3.2 OT benefits semantic verbal fluency in older adults

**Fig. 2.**
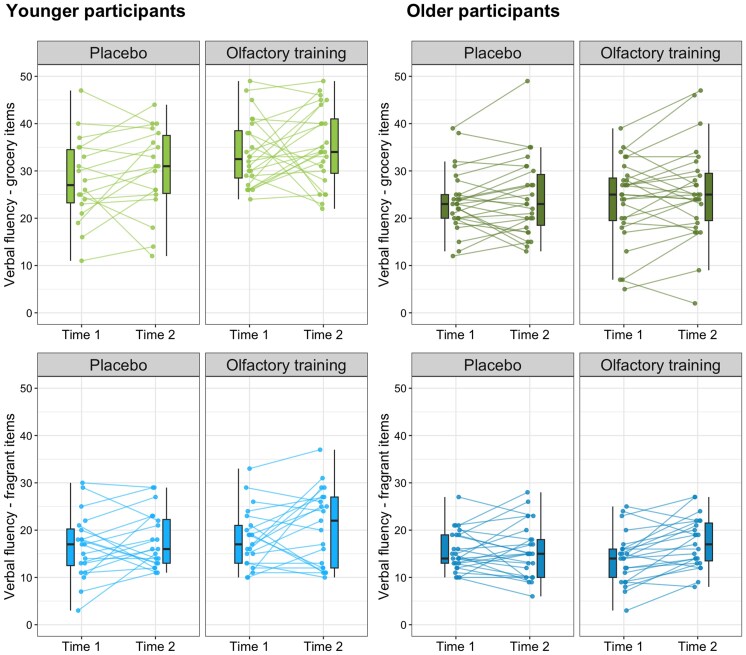
Semantic verbal fluency scores in OT and PGs at pre- and post-training. *P < 0.05, **P < 0.01. Solid lines within boxplots depict medians; rectangles within boxplots depict means.

The omnibus model integrating factors of time, experimental group, age group, and semantic category on semantic verbal fluency (for full results for the model, see Supplementary Tables S1 and S2) revealed a significant age × semantic category interaction (*F*_1,176_ = 6.09, *P* = 0.015, $\eta_{\mathrm{partial}}^{2}=0.033$). Thus, we ran RMA models separately for young adults and older adults groups. The results for the 2 groups separately are presented in Fig. 2.

#### 3.2.1 Older adults

In older adults, there was a significant main effect of time (*F*_1,97_ = 8.50, *P* = 0.004, $\eta_{\mathrm{partial}}^{2}=0.081$). Older adults performed better by listing more items post-training (*M* = 20.7 ± 0.76) than at pretraining (*M* = 19.25 ± 0.65). The main effect of the semantic category (*F*_1,97_ = 42.36, *P* < 0.001, $\eta_{\mathrm{partial}}^{2}=0.304$) indicated that overall older adults listed more grocery store items (*M* = 24.27 ± 0.91) than fragrant items (*M* = 15.65 ± 0.96). The interaction of time × experimental group was significant (*F*_1,97_ = 4.02, *P* = 0.048, $\eta_{\mathrm{partial}}^{2}=0.040$). Post hoc analyses revealed that older adults from the OTG listed more objects post-training (*M* = 21.38 ± 1.08) than pretraining (*M* = 18.99 ± 0.93) (*P* < 0.001), regardless of semantic category, while in the PG, the number of listed items was similar pretraining (*M* = 19.51 ± 0.91) and post-training (*M* = 19.95 ± 1.06; *P* = 0.518). The time × semantic category (*F*_1,97_ = 0.23, *P* = 0.631, $\eta_{\mathrm{partial}}^{2}=0.002$), experimental group × semantic category (*F*_1,97_ = 0.06, *P* = 0.814, $\eta_{\mathrm{partial}}^{2}=$  0.001), and time × experimental group × semantic category (*F*_1,97_ = 1.72, *P* = 0.193, $\eta_{\mathrm{partial}}^{2}=0.017$) interactions were not significant, as well as the main effect of the experimental group (*F*_1,97_ = 0.12, *P* = 0.734, $\eta_{\mathrm{partial}}^{2}=0.001$).

#### 3.2.2 Regression to the mean effect in older adults

Regarding the possibility of the regression to the mean effect in older adults, an ANCOVA has shown a trend-level significance value of the main effect of the experimental group (*F*_1,97_ = 3.82, *P* = 0.053, $\eta_{\mathrm{partial}}^{2}=0.04$), with OTG listing more objects (*M* = 21.82 ± 0.69) than PG (*M* = 19.93 ± 0.68), regardless of semantic category. The adjusted Blomqvist value was *β* = −0.015, *P* = 0.15, SE = 0.15 (thus the confidence interval included zero). Four participants in the OTG and 2 participants in PG exhibited a significant improvement according to the RCI value being >1.96. When treating RCI as a continuous variable, we observed a trend toward higher RCI values in OTG (*M* = 0.54, SD = 1.17) than in the PG (*M* = 0.11, SD = 1.11), *t*(98.5) = 1.93, *P* = 0.057.

#### 3.2.3 Young adults

In the young adults, there was a significant main effect of time (*F*_1,79_ = 4.29, *P* = 0.042, $\eta_{\mathrm{partial}}^{2}=0.051$) on semantic verbal fluency. During the post-test (*M* = 25.95 ± 0.87) young adults listed more words than during the pretest (*M* = 24.15 ± 0.80). There was also a main effect of experimental group (*F*_1,79_ = 6.87, *P* = 0.010, $\eta_{\mathrm{partial}}^{2}=0.080$), with OTG (*M* = 26.93 ± 0.97) listing more items than PG (*M* = 23.17 ± 1.06) at both measurements (*P* = 0.010). There was also a significant effect of semantic category (*F*_1,79_ = 87.68, *P* < 0.001, $\eta_{\mathrm{partial}}^{2}=0.526$). Adults who listed grocery items (*M* = 31.77 ± 1.01) performed better on the task than adults who listed fragrant items (*M* = 18.33 ± 1.02). The time × experimental group (*F*_1,79_ = 0.04, *P* = 0.842, $\eta_{\mathrm{partial}}^{2}=0.001$), time × semantic category (*F*_1,79_ = 0.02, *P* = 0.898, $\eta_{\mathrm{partial}}^{2}<0.001$), experimental group × semantic category (*F*_1,79_ = 1.18, *P* = 0.280, $\eta_{\mathrm{partial}}^{2}=0.015$), and time × experimental group × semantic category (*F*_1,79_ = 0.31, *P* = 0.577, $\eta_{\mathrm{partial}}^{2}=0.004$) interactions were not significant.

## 4. Discussion

This study tested whether OT benefits working memory and semantic verbal fluency in subjectively healthy older adults. The results were compared with a reference group of young adults. Older healthy adults from the OTG listed more words post-training than pretraining, regardless of the semantic category. This effect was not observed in the PG. We did not observe similar effects regarding working memory for digits. As expected, there were no observed effects of OT in the young adults' group. This result is consistent with previous findings, as OT has been shown to be effective in individuals with either olfactory or cognitive deficiencies, including aging as an underlying cause (Schriever et al. 2014; Wegener et al. 2018; Pieniak et al. 2022). The observed effect is small and driven mainly by the individuals with low baseline scores. Furthermore, the presented evidence does not allow us to reject the possibility that lower baseline scores are regressing to the mean rather than improving as a result of OT. The study urges attention toward clinical groups of older people with compromised semantic verbal fluency.

In both age groups, participants listed more grocery than fragrant items. Most people have a rather limited odor-related lexicon and have difficulty in naming even familiar smells, when compared with visual objects (Majid 2021). Some explanation for this phenomenon is attributed to the complexity of the odor-language neural system and a loss of signal quality across its stages (Olofsson and Gottfried 2015). This issue is also dependent on culture, as people using numerous languages, mainly in Asia-Pacific, Americas, and Africa, use extensive categories and terms to describe smells (Majid 2015). For instance, Jahai speakers of the Malay Peninsula can name odors as easily as colors (Majid and Burenhult 2014). It is thus also possible that in cultures with a weak odor-related lexicon, the sense of smell is not seen as particularly important by most people. We did not observe a different outcome of the OT depending on semantic category, which does not support our hypothesis that OT effects on semantic verbal fluency in older adults may be domain-specific. However, inconsistent results of previous studies, partially arising from diverse methodological approaches, urge further empirical verification. The first study to report improvement in semantic verbal fluency in older adults did not specify the target semantic category (Wegener et al. 2018). Further studies proposed listing words within a given semantic category such as grocery items (Oleszkiewicz et al. 2021a), but the current study for the first time investigated odor-specific words. While preliminary evidence for the cross-modal transfer of OT effects exists (Olofsson et al. 2020), further research is needed to more systematically address this problem.

Noteworthy, the OT compliance in the older adults group exceeded 90%, significantly more than in the young adults cohort. OT performance in the older adults group far exceeds the conventional 50% threshold for inclusion in the study. It may result from the overall conscientiousness of older adult participants and their commitment to the study, but it also pinpoints the positive attitude toward the OT. From the practical point of view, this is an important argument in favor of introducing OT in clinical and psychological settings. Older adults who are advised to perform OT to maintain olfactory health or psychological well-being will likely engage in OT without much encouragement or monitoring.

Older adults group had an overrepresentation of women. Women have better olfactory abilities than men (Sorokowski et al. 2019), value olfaction more (Croy et al. 2010), and are more attentive and interested in surrounding odors (Oleszkiewicz et al. 2021b; Rokosz et al. 2024), which may explain why we observed an effect of OT on odor-related semantic verbal fluency. Further studies among aging populations should be conducted with a more balanced sex ratio to replicate these findings in men.

Higher scores obtained during the post-OT measurement, irrespective of group, suggest the occurrence of the learning effect. It could have been mitigated by using parallel versions of the tests at each measurement. However, our study shows that changing the semantic category can significantly change individual performance within a given task, making it impossible to compare individual pre- and post-OT results. In studies testing the effectiveness of interventions, there is a possibility of significant results being due to random factors attributing to regression to the mean effect (Senn 2006). We tested 3 methods assessing this concern (Blomqvist 1977; Jacobson and Truax 1991; Senn 2006); they however seem inconclusive, as the effects are very close to the marginal points and cannot clearly answer this question, which aids to the need of approaching our results with caution. Additionally, despite our best efforts to randomize the participants, we noted that the OTG adult group generated more grocery items than the PG adult group in both measurements. Since the 2 groups were sex and age-balanced, we cannot explain this effect other than by random causes. In the older adults group, we observed that quitting the study was related to a more advanced age, possibly due to fatigue or the reluctance to engage in a longitudinal study. The oldest participants could also not return for the second measurement because of the change in their health, but our study did not monitor these reasons. On the other hand, older adults had greater OT compliance than the adults, and this might explain why we observed a significant effect on semantic verbal fluency only in the elderly group. Even with a robust sample and high OT compliance, the effect was small. Therefore, OT should be seen rather as a supportive method to prevent cognitive deterioration in elderly women. Nonetheless, it is an enjoyable, easy, and cost-efficient method that participants eagerly incorporate into their daily routine. However, measures have to be taken to ensure OT completion of the training by distinctively aged participants. All the above conclusions have to be interpreted with caution and mainly apply to elderly women.

## 5. Conclusion

Our study shows that OT marginally benefits semantic verbal fluency in healthy aging adult populations. This effect does not seem to be domain-specific. Conclusions are mainly limited to older women, as older men are less eager to recruit for intervention studies.

## Supplementary Material

bjaf032_Supplementary_Data

## Data Availability

Data are available in the Supplementary materials. Code for the mobile app used to monitor compliance and guide participants through the OT session can be shared on request.
