# Supplementary material for: Olfactory training enhances semantic verbal fluency in healthy older adults, but only for individuals with low baseline performance
Source: Chem Senses. 2025 Sep 5;50:bjaf032. doi: 10.1093/chemse/bjaf032 (PMC12510804; doi:10.1093/chemse/bjaf032)
Supplement: bjaf032_Supplementary_Data [file bjaf032_supplementary_data.zip › Supplementary Material.docx]

**Supplementary Material**

**Supplementary Table S1**

*Within subjects effects in the verbal fluency model integrating time, group, age, and semantic category.*

**Supplementary Table S2**

*Between subjects effects in the verbal fluency model integrating time, group, age, and semantic category*

**Supplementary Table S1**

*Within subjects effects in the verbal fluency model integrating time, group, age, and semantic category.*

|  | Sum of Squares | Mean Square | **F(1, 176)** | **η²_p_** |
| --- | --- | --- | --- | --- |
| residual | 3601.02 | 20.46 |  |  |
| time | 233.78 | 233.78 | 11.43^***^ | 0.061 |
| time ✻ group | 29.77 | 29.77 | 1.45 | 0.008 |
| time ✻ semantic category | 2.70 | 2.70 | 0.13 | 0.001 |
| time ✻ age | 3.31 | 3.31 | 0.16 | 0.001 |
| time ✻ group ✻ semantic category | 28.55 | 28.55 | 1.40 | 0.008 |
| time ✻ group ✻ age | 14.44 | 14.44 | 0.71 | 0.004 |
| time ✻ semantic category ✻ age | 0.34 | 0.34 | 0.02 | <0.001 |
| time ✻ group ✻ semantic category ✻ age | 0.51 | 0.51 | 0.03 | <0.001 |

****p*<.001

**Supplementary Table S2**

*Between subjects effects in the verbal fluency model integrating time, group, age, and semantic category*

|  | Sum of Squares | Mean Square | ***F*(1, 176)** | **η²_p_** |
| --- | --- | --- | --- | --- |
| residual | 15248.30 | 86.60 |  |  |
| group | 401.80 | 401.80 | 4.64^*^ | 0.026 |
| semantic category | 11008.40 | 11008.40 | 127.06^***^ | 0.419 |
| age | 2345.90 | 2345.90 | 27.08^***^ | 0.133 |
| group ✻ semantic category | 79.40 | 79.40 | 0.92 | 0.005 |
| group ✻ age | 248.40 | 248.40 | 2.87 | 0.016 |
| semantic category ✻ age | 527.40 | 527.40 | 6.09^*^ | 0.033 |
| group ✻ semantic category ✻ age | 35.20 | 35.20 | 0.48 | 0.002 |

**p*<.05; ****p*<.001
